# Metformin alleviates lead-induced mitochondrial fragmentation via AMPK/Nrf2 activation in SH-SY5Y cells

**DOI:** 10.1016/j.redox.2020.101626

**Published:** 2020-06-30

**Authors:** Luoyao Yang, Xiaoyi Li, Anli Jiang, Xintong Li, Wei Chang, Jun Chen, Fang Ye

**Affiliations:** a Department of Occupational and Environmental Health, School of Public Health, Tongji Medical College, Huazhong University of Science and Technology, Wuhan, PR China; b Ministry of Education Key Lab for Environment and Health, School of Public Health, Tongji Medical College, Huazhong University of Science and Technology, Wuhan, PR China; c Center for Translational Medicine, Wuhan Union Hospital, Huazhong University of Science and Technology, Wuhan, PR China; d Department of Public Health, Medical College, Wuhan University of Science and Technology, Wuhan, PR China

**Keywords:** Lead, Mitochondrial fragmentation, Metformin, Nrf2, Oxidative stress

## Abstract

As a widely acknowledged environmental pollutant, lead (Pb) exhibits neurological toxicity primarily due to the vulnerability of neural system. It is suggested that Pb could perturb mitochondrial function, triggering the following disturbance of cellular homeostasis. Here, we focused on the role of mitochondrial dynamics in Pb-induced cell damage. Pb exposure enhanced mitochondrial fragmentation and elevated p-Drp1 (s616) level in a reactive oxygen species (ROS) dependent manner, leading to cell death and energy shortage. By applying metformin, an AMP-activated protein kinase (AMPK) activator, these impairments could be alleviated via activation of AMPK, validated by experiments of pharmacological inhibition of AMPK. Further investigation confirmed that nuclear factor erythroid 2-related factor 2 (Nrf2), a transcription factor managing antioxidative function, and its downstream antioxidant detoxifying enzyme were activated by metformin, resulting in the inhibition of the Pb-caused oxidative stress. Moreover, Nrf2 mediated the protection of metformin against mitochondrial fragmentation induced by Pb exposure, while knockdown of Nrf2 abrogated the protective effect. Finally, the treatment of Mdivi-1, a mitochondrial fission inhibitor, reversed Pb-triggered cell death, revealing that excessive mitochondrial fission is detrimental. To conclude, metformin could ameliorate Pb-induced mitochondrial fragmentation via antioxidative effects originated from AMPK/Nrf2 pathway activation, promoting energy supply and cell survival.

## Highlights

• Pb caused mitochondrial fragmentation in a ROS dependent manner.
• Metformin alleviated Pb-induced mitochondrial fission via Nrf2 activation.
• AMPK mediated metformin-induced Nrf2 activation.
• Inhibition of mitochondrial fragmentation rescued Pb-induced cell death.

## Graphical abstract

**Figure:**
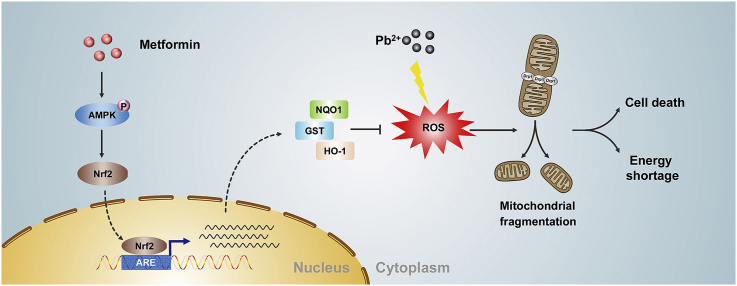

## 1 Introduction

Lead (Pb), a ubiquitously distributed heavy metal, is recognized as an environmental pollutant threatening human health, especially the development of nervous system [1,2]. Studies have confirmed that major contribution to Pb toxicity lay in its capability to disturb mitochondrial homeostasis, resulting in impaired neuron function [3]. However, the detailed mechanism is obscure, and the exploration of which is still ongoing. Recently, mitochondrial dynamics and biogenesis is suggested to be the target of Pb neurotoxicity, the defects of which lead to cell dysfunction and even death [4]. Enhanced mitochondrial fragmentation has been observed in Pb-exposed human neurons and SH-SY5Y cell, inducing the boost in cell death [5,6].

As a dynamic organelle, mitochondria manage to retain the balance of fusion/fission in physiological situations [7]. The coordination of mitochondrial fusion/fission events link closely with mitochondrial function, integrating maintenance of cellular redox state, calcium homeostasis and energy supply [8,9]. In response to stress, mitochondrial dynamics is impaired and further participates in the decision of cell fate [8]. Particularly, accumulating evidence suggests that activation of excessive mitochondrial fission exacerbates oxidative stress and executes programmed cell death [10]. In Alzheimer's disease neurons, a rise in fission-related proteins was observed, indicating the potential impact of mitochondrial fragmentation in neuronal degradation [11]. Accordingly, the boost in mitochondrial fragmentation might be responsible for Pb-induced neuron damage, suggesting that it could be a promising target to diminish Pb-triggered impairment.

Mitochondrial fission is a multifactorial process regulated by various pathways, one of which relating to cellular energy homeostasis is AMP-activated protein kinase (AMPK) pathway [12]. Under condition of energy shortage, AMPK is reported to be activated, inducing mitochondrial fission and mitophagy [13]. However, in study of Empagliflozin-ameliorated diabetic cardiac microvascular injury, activation of AMPK induces mitochondrial fission via dynamin-related/-like protein 1 (Drp1) phosphorylation and activation, which is a critical modulator to stimulate mitochondrial fission [14]. Another research reveals that protective effect of metformin, an AMPK activator, against atherosclerosis in diabetes was based on AMPK-inhibited mitochondrial fission [15]. The role of AMPK in mitochondrial fusion/fission is complicated, and how AMPK pathway regulates Pb-triggered mitochondrial fission is in lack of evidence and requires further exploit.

Serving as a stress sensor, AMPK exerts beneficial effect in prevention of ROS accumulation to alleviate oxidative stress [16,17]. Notably, AMPK pathway shares distinct crosstalk with antioxidant response, one master regulator of which is nuclear factor erythroid 2-related factor 2 (Nrf2) [18]. On sensing redox system imbalance, Nrf2 is activated to upregulate antioxidant gene expression [19]. Prior studies confirmed that pharmacological activation of AMPK alleviates oxidative stress in both human cardiomyocytes and neural stem cells [20,21]. The underlying mechanism is demonstrated to be the induction of AMPK on Nrf2 and its downstream target heme oxygenase-1 (HO-1) [22,23]. Recently, the direct phosphorylating effect of AMPK on Nrf2 has been identified [24]. Based on our previous investigation on the contribution of Nrf2/HO-1 signaling pathway to the recovery of Pb-induced oxidative stress [25,26], we postulate that AMPK plays a regulatory role in the antioxidant defense. Additionally, metformin was reported to render reno-protection against Pb nephrotoxicity in rats [27]. Here, we investigated whether metformin-triggered AMPK pathway participates in antioxidant response upon Pb exposure. Considering the close linkage between mitochondrial dysfunction and neurological impairment, we explored the role of mitochondrial fragmentation in Pb-induced cell damage, and whether it can be reversed by metformin.

## 2 Materials and methods

### 2.1 Cell culture

Human neuroblastoma SH-SY5Y cells, obtained from the American Type Culture Collection (CA, USA), were cultured in DMEM/F12 medium supplemented with 10% fetal bovine serum (FBS), 1% unessential amino acids, 100 U/mL penicillin/streptomycin at 37 °C in a humidified 5% CO_2_ atmosphere. The cells we received were 24 passages, and the 29˗36 passages were used in current study.

### 2.2 Cell viability

Cell viability was assessed with the Cell Counter Kit 8 (CCK-8) assay (Dojindo Laboratories., Japan) according to the manufacturer's instruction. SH-SY5Y cells were seeded on 96-well plates and left to attach overnight. After treatments, 10 mM of the CCK-8 solution was dissolved by serum free DMEM/F12 medium and added to each well of the plate. Then, cells were incubated in the incubator for 1 h, and the absorbance at 450 nm was quantified by automated microplate reader (Synergy 2, BioTec, CA, USA).

### 2.3 Immunofluorescence

SH-SY5Y cells were plated on glass cover slips into 24-well plates and left to attach overnight. After the indicated treatments, cells were fixed for 30 min in 4% paraformaldehyde solution, permeabilized by 0.1% triton-100, then blocked with 10% goat serum for 30 min. Cells were incubated overnight with Nrf2 (1:50) or Tom20 (1:100) antibody at 4 °C. Cover slips were washed with phosphate-buffered saline (PBS) (5 min, 3 times) and incubated with anti-mouse Alexa Fluor 488 (1:200) for 1 h at room temperature protected from light. Finally, the cover slips were stained with 0.1% DAPI for 5 min. The images were acquired by a fluorescence microscope (Olympus, Japan) or confocal microscope (A1, Nikon, Japan). The mitochondrial morphology in images within a resolvable 225 μm^2^ per region of interest (ROI), which was peripheral to the microtubule-organizing center, was assessed using various tools in ImageJ (National Institutes of Health). The number of mitochondrial fragments and the area of each fragment per ROI were obtained and analyzed [28].

### 2.4 Western blot

Whole cell proteins were extracted using ice-cold RIPA buffer (Beyotime Institute of Biotechnology, Ltd., Shanghai, China) containing protein inhibitor cocktail (Roche, Germany). Protein concentrations were determined by BCA protein assay kit (Pierce, Rockford, IL, USA) as described by the manufacturer. Western blot was applied to detect the proteins of Drp1, p-Drp1, mitofusin 1 (Mfn1), Nrf2, HO-1, AMPK, p-AMPK, Raptor, p-Raptor and β-actin shown in sTable 1. The density of all immunoreactive bands was analyzed by Gel-pro software (Media Cybernetics, USA).

### 2.5 Electrophoretic mobility shift assay (EMSA)

The Nuclear and Cytoplasmic Protein Extraction Kit (Beyotime Institute of Biotechnology, Ltd., Shanghai, China) was used to prepare the nuclear proteins, SH-SY5Y cells were first lysed by cytoplasmic protein lysis buffer and incubated for 10 min at 4 °C. The lysates were ultracentrifuged at 12,000 g for 5 min at 4 °C, and the supernatants were collected as cytoplasmic proteins. The pelleted nuclei were resuspended in nuclear protein lysis buffer and incubated for 10 min at 4 °C, then the lysates were centrifuged, and the supernates containing the nuclear proteins were obtained and the protein concentrations were determined. Synthetic double-strand oligonucleotide (Beyotime Institute of Biotechnology, Ltd., Shanghai, China) containing the Nrf2 binding domain anti-oxidative response element (ARE) was labeled with biotin. The sequence is 5-ACT GAG GGT GAC TCA GCA AAA TC-3, 3-TGA CTC CCA CTG AGT CGT TTT AG-5. Hemiluminescent Nucleic Acid Detection Module Kit (Pierce, Rockford, IL, USA) was applied to determine Nrf2-ARE binding activity. Approximately 7.5 μg of the nuclear extract was incubated on ice for 15 min in gel shift binding buffer. DNA-protein complexes were resolved by 6.5% polyacrylamide gel electrophoresis at 100 V for 1 h, and then transferred to a nylon membrane. After crosslinking for 10 min by UV-light instrument, the membrane was blocked, conjugated, washed, and substrated by a corresponding solution. Finally, the resultant signal was visualized using an enhanced ECL chemiluminescence kit (Pierce, Rockford, IL, USA).

### 2.6 DCFH and Calcein-AM/PI/Hochest 33342 staining

SH-SY5Y cells (3 × 10^5^/mL) were seeded into 6-well plates. After indicated treatments, the medium was replaced by fresh serum-free medium containing fluorescence probe 2′7′-dichlorodihydrofluorescein diacetate (DCFH-DA) (Beyotime Institute of Biotechnology, Ltd., Shanghai, China) or Calcein-AM/PI/Hochest 33342 (Invitrogen) for 30 min at 37 °C in the dark and then washed 3 times with serum-free medium. The images were acquired by fluorescence microscope. The mean fluorescence of DCFH-DA was calculated; the percent of PI^+^ cells, representing dead cells, were analyzed.

### 2.7 siRNA transfection

Pre-designed siRNA for human Nrf2 (sc-37030) and negative control siRNA (sc-37007) were obtained from Santa Cruz (CA, USA) and transfected into cells by Lipofectamine RNAi Max (Invitrogen) according to the protocol. For six-well plates, siRNA (60 pmol) was incubated with transfection reagent in Opti MEM (Invitrogen) for 10 min at room temperature to allow the formation of transfection complexes. The cells were washed twice by Opti MEM, and then the transfection complexes were dropped into wells containing OptiMEM (1 ml). After 8 h of transfection, cells were changed to fresh medium containing 10% FBS and then subjected to various treatments as described.

### 2.8 ATP assay

The cellular ATP levels were detected by an ATP Bioluminescence Assay Kit (Beyotime Biotechnology Co., China). Cells were seeded in 6-well plates and left overnight. After treatment, cells were lysed and centrifuged at 12 000 rpm for 5 min at 4 °C and the supernatants were collected. Working solution (100 μL) was added to 20 μL of sample in the 96-well plate, and the luminescence was measured immediately on an automated microplate reader. Measurements from all samples were normalized to protein concentration.

### 2.9 Glutathione (GSH) and Na^+^-K^+^-ATPase assay

The cells were seeded in plates and left overnight. After the indicated treatment, sonication was carried out. Total amount of 100 μL of the sonicated cell suspension was collected and centrifuged at 3500 rpm for 10 min to acquire supernatant. For GSH detection (A006-2-1, Jiancheng, Nanjing, China), reaction solution of GSH was added and incubated for 5 min before the absorbance at 405 nm was detected. For Na^+^-K^+^-ATPase measurement (A070-2-2, Jiancheng, Nanjing, China), reaction solution of GSH was added and incubated for 5 min at room temperature before the absorbance at 636 nm was detected. Measurements of all samples were normalized to protein concentration.

### 2.10 Lactic dehydrogenase (LDH) leakage assay

LDH leakage assay (Beyotime Biotechnology, China) was performed according to the manufacturer's instructions. After treatment, the 96-well plate was centrifuged at 200 rpm for 5 min. Subsequently, 100 μL of the cell supernatant was transferred to a fresh 96-well plate, and 100 μL of the reaction solution was added and incubated for 30 min at 37 °C before the absorbance at 490 nm was detected.

### 2.11 Transmission electron microscopy

After treatment, 1 × 10^7^ SH-SY5Y cells were fixed by 2.0% glutaraldehyde in 0.1 M sodium cacodylate buffer (pH = 7.4) for 1 h at room temperature and scraped by using cell scraper. Ultrathin osmium-stained SH-SY5Y cell sections were prepared and imaging was performed with a Tecnai ™ (FEI, USA) electron microscope.

### 2.12 Real-time PCR

RNA was extracted from SH-SY5Y with TRIzol reagent (Invitrogen, Carlsbad, CA, USA) according to the manufacturer's protocol. The quality and quantity of RNA were detected by NanoDrop 1000 (Thermo Scientific, USA). Approximately 2 μg of RNA was used for cDNA synthesis with the Ominiscript RT Kit (Thermo Scientific, USA). RT-PCR was performed in 10 μL containing 100 nM primers (Table 1) purchased from Invitrogen (CA, USA) and SYBR Green PCR Master Mix (Life Technology, CA, USA) by ABI 7900HT (Life Technology, CA, USA). The mRNA levels of all samples were normalized to human GAPDH mRNA expression by the comparative cycle threshold method. At the end of amplification, the identity and purity of the amplified product was checked by analyzing the melting curve.

**Table 1:** Primers used in current study.

Gene	Primer sequence		Amplicon (bp)
Drp1	F	5′-AAT CGT CGT AGT GGG AAC GC-3′	170
	R	5′-TCC ACC CCA TTT TCT TCT CCT-3′	
Fis1	F	5′-GTA AAG GCA TCG TGC TGC TC-3′	82
	R	5′-ACG GCC AGG TAG AAG ACG TA-3′	
GCLC	F	5′-CAA GGA CGT TCT CAA GTG GG-3′	170
	R	5′-CAT ACT CTG GTC TCC AAA GG-3′	
GST-α1	F	5′-AGC CCA AGC TCCACT ACT TC-3′	84
	R	5′-CTT CAA ACT CTA CTC CAG CTG C-3′	
HO-1	F	5′-AAC TTT CAG AAG GGC CAG GT-3′	112
	R	5′-CTG GGC TCT CCT TGT TGC-3′	
Mfn1	F	5′-GCC TCC TCT CCG CCT TTA ACT T-3′	150
	R	5′-GCC TTC TTA GCC AGC ACA AAG-3′	
NQO1	F	5′-CGC AGA CCT TGT GAT ATT CCA G-3′	135
	R	5′-CGT TTC TTC CAT CCT TCC AGG-3′	
Nrf2	F	5′-ATT GCC TGT AAG TCC TGG TCA-3′	182
	R	5′-ACT GCT CTT TGG ACA TCA TTT CG-3′	
Opa1	F	5′-CGG GAA CTT GAC CGG AAT GA-3′	168
	R	5′-CGC AGC TGG AAG GTA GAT GT-3′	
GAPDH	F	5′-CTG ACT TCA ACA GCG ACA CC-3′	114
	R	5′-TGC TGT AGC CAA ATT CGT TGT -3′	

### 2.13 Statistical analysis

All experiments were repeated at least thrice. Data were expressed as mean ± SEM. The results were analyzed by one-way ANOVA with post hoc Dunnett's test or LSD's test. A *p* value of <0.05 was taken to be significant.

## 3 Results

### 3.1 Metformin alleviated Pb-induced cell death and energy shortage

**Fig. 1:**
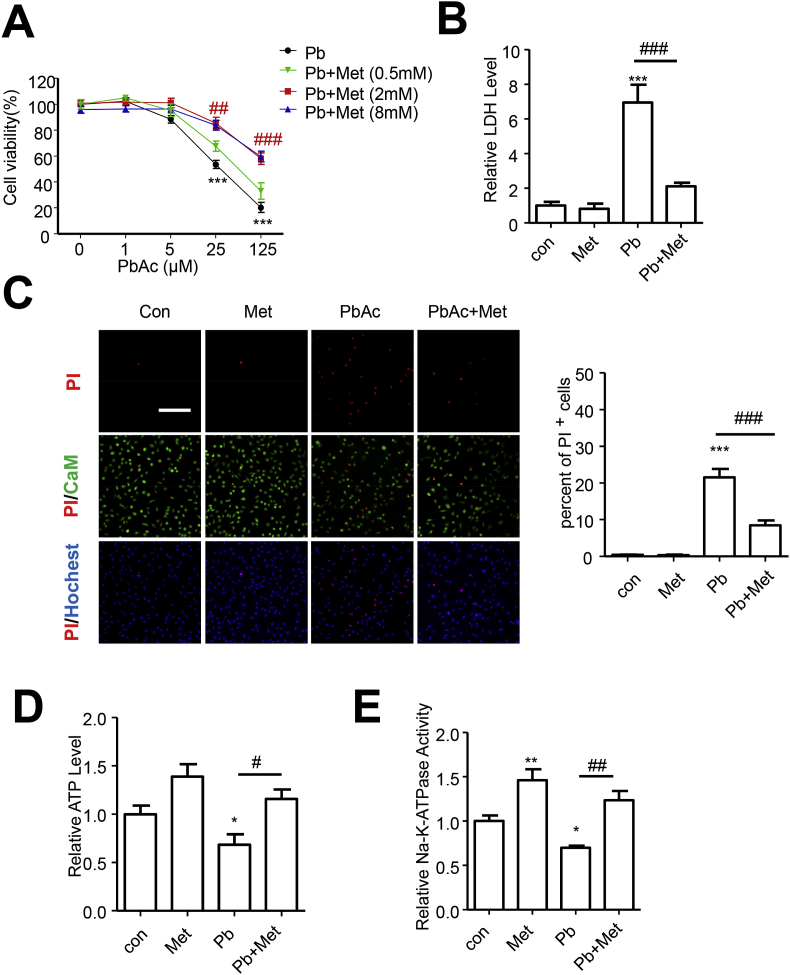
Metformin alleviated Pb-induced cell death and energy shortage. (A) SH-SY5Y cells pretreated with metformin (0.5, 2 or 8 mM) for 6 h were exposed to various dose of PbAc (1, 5, 25 or 125 μM) for 24 h, cell viabilities were analyzed by CCK8 assay, n = 5. (B–E) Cells were exposed to PbAc (25 μM) after 6 h treatment of metformin (2 mM), LDH release in medium were detected, n = 4 (B), and CaM/PI/Hochest33342 were used to analyzed cell death (Scale bar = 500 μm, n = 6) (C); ATP content (D) and Na^+^-K^+^-ATPase activity (E) were detected, n = 3. *P < 0.05 and ***P < 0.001 represent significant differences compared with the untreated cells and #P < 0.05, ##P < 0.01 and ###P < 0.001 represent significant differences between groups with or without metformin pretreatment exposed to PbAc.

Firstly, the dose-dependent effect of lead acetate (PbAc) on cell viability was attenuated by metformin efficiently at concentration of 2 mM and beyond (Fig. 1A). Metformin treatment solely showed no capability in reducing cell viability even at high concentration of 8 mM (sFig. 1). Thus, in the following research, concentration of 25 μM and 2 mM was chosen for PbAc and metformin treatment respectively. The leakage of LDH in medium, an indicator of cell death, dramatically decreased after metformin administration under condition of PbAc treatment (Fig. 1B). Results from Calcein-AM/PI/Hochest 33342 staining also showed that metformin significantly reduced the percent of PbAc-induced dead cells, represented by PI^+^, from 20% to 8% (Fig. 1C). In addition, a significant decrease was observed in measurement of ATP level and Na^+^-K^+^-ATPase activity after PbAc exposure, which was eliminated by metformin (Fig. 1D–E). These data suggested that PbAc exerted a detrimental impact on cell survival and energy balance, which could be intensively abolished by metformin.

### 3.2 Pb-induced mitochondrial fission could be inhibited by metformin

**Fig. 2:**
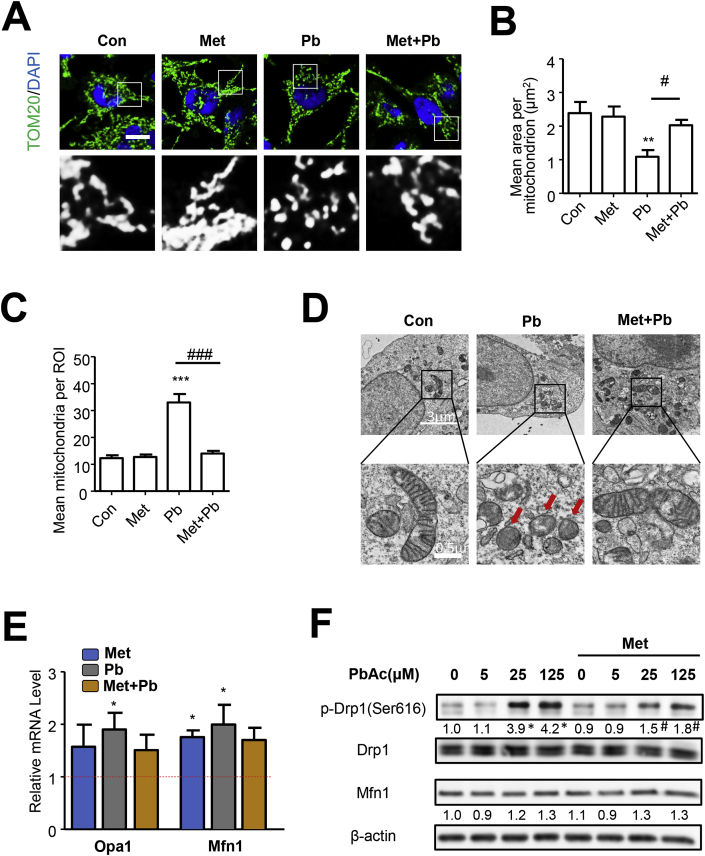
Pb-induced mitochondrial fission could be inhibited by metformin. (A–D) SH-SY5Y cells pretreated with metformin (2 mM) for 6 h were exposed to PbAc (25 μM) for 24 h, mitochondrial morphology was analyzed by immunofluorescence of TOM20, scale bar = 10 μm, n = 25 (A–C); transmission electron microscopy was performed to investigate the ultrastructure of mitochondria, red arrows marked the fragmented mitochondria (D). (E) After treatment, the mRNA level of fusion associated proteins (Opa1 and Mfn1) were analyzed, n = 3. (F) Cells pretreated with metformin were treated with PbAc for 24 h, the protein levels were detected by western blot, n = 3. *P < 0.05, **P < 0.01 and ***P < 0.001 represent significant differences compared with the untreated cells and #P < 0.05 and ###P < 0.001 represent significant differences between groups with or without metformin pretreatment exposed to PbAc. (For interpretation of the references to colour in this figure legend, the reader is referred to the Web version of this article.)

Previous study suggested that Pb treatment impaired mitochondrial functions, where the disturbance of mitochondrial fusion-fission balance plays a crucial role [5]. In present study, mitochondrial morphology was determined by immunofluorescence of Tom20, a protein located in outer mitochondria membrane. SH-SY5Y cells treated with PbAc by 24 h exhibited distinct mitochondrial fragmentation, which could be partially inhibited by metformin (Fig. 2A–C). In addition, results from transmission electron microscopy detecting cellular ultrastructure demonstrated that mitochondria were shattered, swelled and ruptured in cells exposed to PbAc, while metformin treatment could obviously reverse the damage caused by PbAc (Fig. 2D). Hereafter, the mRNA levels of proteins associated with mitochondrial dynamics, including Drp1, Fis1, Opa1 and Mfn1 were detected. PbAc treatment could upregulate Opa1 and Mfn1, which play crucial roles in promoting mitochondrial fusion, but not the fission associated protein Drp1 and Fis1 (Fig. 2E and sFig. 2). However, protein analysis showed that level of p-Drp1 (Ser616) was increased but Mfn1 did not change at all after PbAc treatment, which was inconsistent with PCR results. Drp1 acts as a key protein mediating mitochondrial fission, and the activity of which can be regulated by post-translational modification. It is well established that phosphorylation at serine 616 would promote mitochondrial fission [8]. Here, metformin treatment inhibited the Pb-induced p-Drp1 (s616) upregulation (Fig. 2F). These results confirmed that Pb-triggered mitochondrial fragmentation can be eliminated by metformin, where p-Drp1 is possibly involved.

### 3.3 Oxidative stress mediated Pb-induced mitochondrial fragmentation

**Fig. 3:**
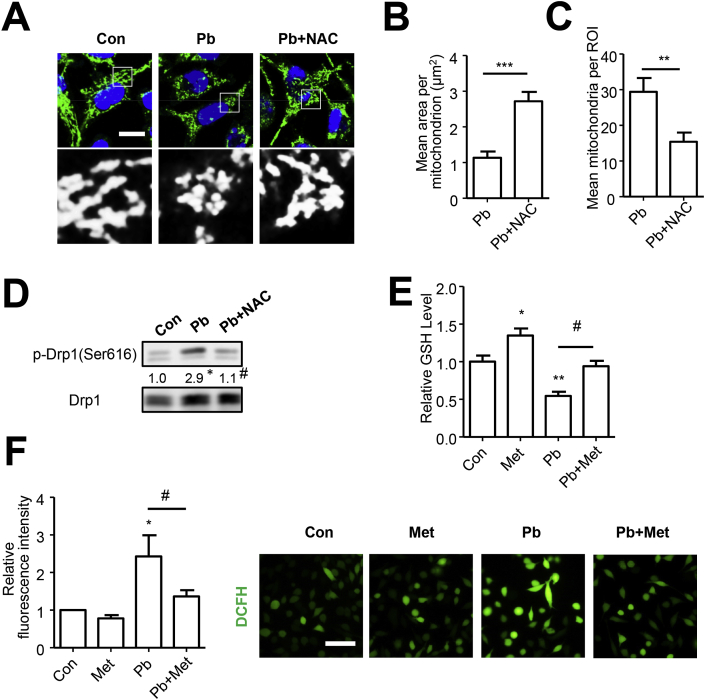
Metformin inhibited PbAc-induced oxidative stress, which mediated mitochondrial fragmentation. (A–D) SH-SY5Y cells pretreated with NAC (5 mM) were exposed to PbAc for 24 h, the immunofluorescence of TOM20 were performed and the mitochondrial morphology was analyzed, scale bar = 10 μm, n = 30 (A–C); the levels of p-Drp1 (s616) and Drp1 were detected, n = 3 (D). (E–F) GSH content (n = 4) and cellular ROS level (scale bar = 50 μm, n = 6) were analyzed in cells treated with metformin and PbAc. *P < 0.05, **P < 0.01 and ***P < 0.001 represent significant differences compared with the untreated cells and #P < 0.05 represents significant differences between groups with or without metformin/NAC pretreatment exposed to PbAc.

As oxidative stress is an important factor involved in mitochondrial fission process and can be induced by Pb exposure [29], efforts were made to explore the role of oxidative stress in Pb-induced mitochondrial fragmentation. The treatment of 5 mM N-acetyl-l-cysteine (NAC), a GSH precursor, or 0.5 mM Tempol, superoxide dismutase (SOD)-mimetic drug which both could efficiently neutralizes ROS, intensively rescued mitochondria from PbAc-induced fragmentation and abolished the enhancement of phosphorylated Drp1 levels (Fig. 3A–D and sFig. 3A–D).

### 3.4 Metformin rescued Pb-induced oxidative stress and mitochondrial fission depending on Nrf2 activation

Metformin has been reported to exert antioxidant effect [16], which was also detected in response to Pb-induced oxidative stress in present study. As shown in Fig. 3E-F, the ROS accumulation detected by DCFH fluorescent probe in PbAc-exposed SH-SY5Y cells could be mitigated by metformin treatment, which can also augment GSH content. These results suggested that metformin was capable of attenuating Pb-induced ROS accumulation and antioxidant response suppression.

**Fig. 4:**
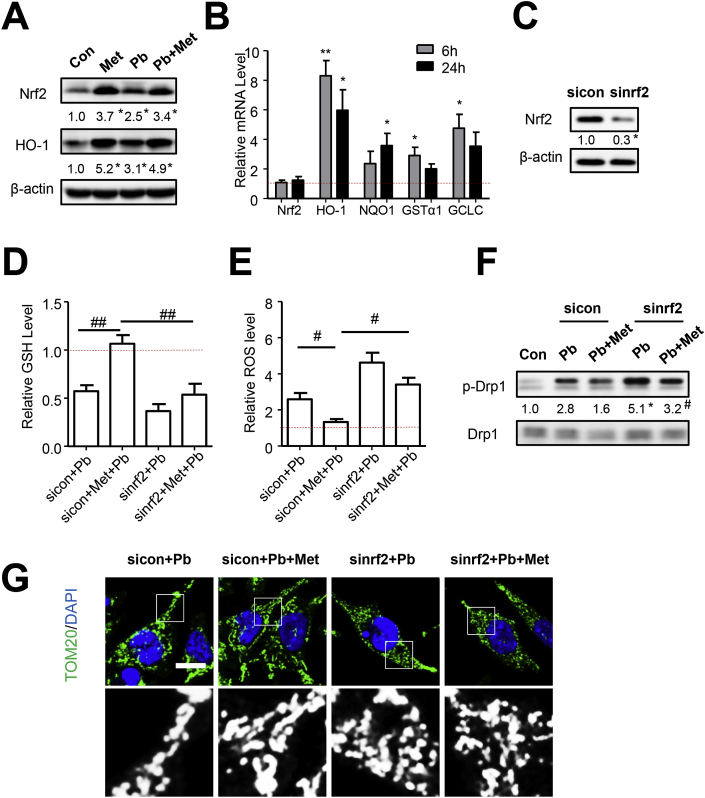
Metformin rescued Pb-induced mitochondrial fission depending on Nrf2 activation. (A) Nrf2 and HO-1 proteins were detected in cells treated with metformin or PbAc for 24 h, n = 3. (B) The mRNA levels of Nrf2, HO-1, NQO1, GST-α1 and GCLC were analyzed in metformin-treated cells, n = 3. (C) SiRNA transfection were performed to knock down Nrf2. (D–E) Nrf2-knockdown cells treated with metformin were exposed to PbAc for 24 h, GSH content (D) and cellular ROS level (E) were detected. (F–G) After treatment, p-Drp1/Drp1 and mitochondrial morphology (n = 25) were analyzed, scale bar = 10 μm *P < 0.05 and **P < 0.01 represent significant differences compared with the untreated cells; #P < 0.05 and ##P < 0.01 represent significant differences between indicated groups.

**Fig. 5:**
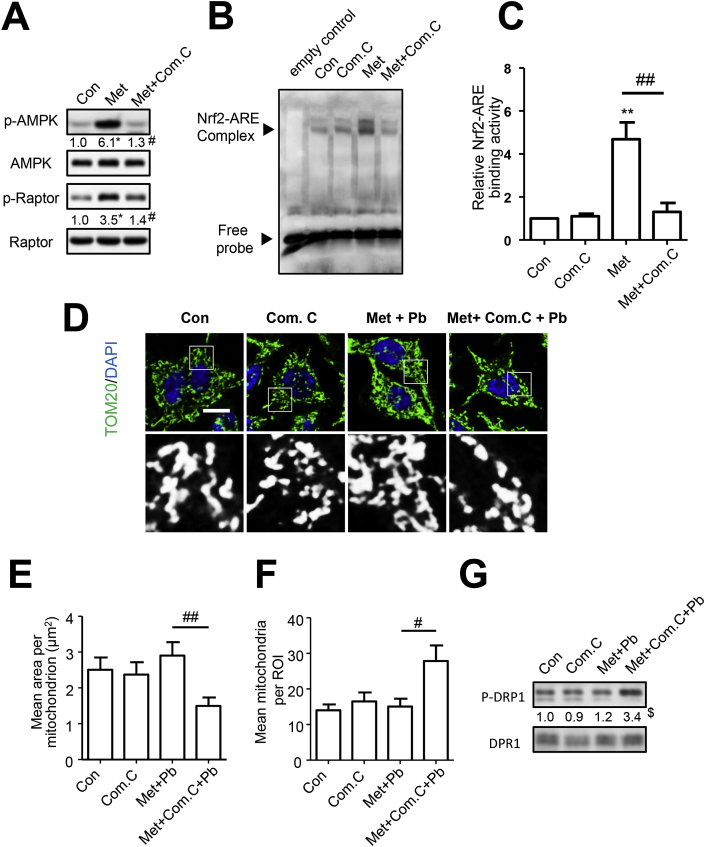
AMPK mediated metformin-induced Nrf2 activation. (A–C) SH-SY5Y cells were treated with compound C (10 μM) and metformin for 6 h, the proteins were detected by western blot, n = 3 (A); EMSA assay was applied to analyze the Nrf2-ARE binding activity, n = 3 (B–C). (D–G) After treatment of Com.C (10 μM) and metformin, cells suffered from PbAc exposure for 24 h, the mitochondrial morphology (n = 25) (D–F) and p-Drp1/Drp1 (G) were analyzed. Com.C represents compound C. *P < 0.05 represents significant differences compared with the untreated cells; #P < 0.05 and ##P < 0.01 represent significant differences between indicated groups.

Nrf2 is accepted as the main regulator to retrieve redox balance in defense of oxidative stress [30]. Our previous work has identified the protective role of Nrf2 against oxidative stress and cell death caused by PbAc [26]. In the present study, we examined the effect of metformin on Nrf2 activation. Metformin increased nuclear levels of Nrf2 (Fig. 4A and sFig. 3A) and elevated the Nrf2-ARE binding activity (Fig. 5B–C). By detecting mRNA levels, the activation of Nrf2 downstream genes, including HO-1, NQO-1, GST*α*1 and GCLC, were observed (Fig. 4B). However, the Nrf2 mRNA level was not increased, suggesting that metformin might activate Nrf2 via post-translational modification. Consistent with our previous study [26],Nrf2/HO-1 pathway could be activated by PbAc exposure by cellular self-defense mechanism, but treatment with metformin did not potentiate Pb in activation of Nrf2 signaling. To investigate the role Nrf2 played in metformin rescued oxidative stress and mitochondrial fission caused by Pb, the siRNA transfection was successfully applied to knock down Nrf2 expression (Fig. 4C). As shown in Fig. 4D, the elevation of GSH by metformin was absent in partial loss of Nrf2. Supporting this data, metformin-triggered inhibition of ROS accumulation was not observed in Nrf2 knockdown cells (Fig. 4E). These results indicated that metformin responded to Pb-induced oxidative insults in a Nrf2-dependent manner. Considering the linkage between mitochondrial dynamics and redox balance, we further assessed mitochondrial fission in Nrf2 knockdown cells. Nrf2 knockdown abrogated the alteration of metformin on Pb-induced activation of phosphorylated Drp1 (Fig. 4F). Additionally, metformin lost the capacity to reverse Pb-induced mitochondrial fragmentation in response to Nrf2 knockdown (Fig. 5G and sFig. 4). Taken together, metformin rescued Pb-induced oxidative stress and mitochondrial fission is Nrf2-dependent.

### 3.5 AMPK mediated metformin-induced Nrf2 activation

The maintenance of cellular redox state and energy metabolism link closely together, due to the involvement of overlapping AMPK/Nrf2 pathway [31,32]. Prior studies confirmed the stimulatory effect of AMPK on Nrf2 pathway [32,33]. It is conceivable that metformin rescued Pb-induced redox state disturbance and subsequent mitochondrial fission through AMPK/Nrf2 signaling pathway. After the application of Compound C, an AMPK inhibitor, the augment in p-AMPK level by metformin was abolished (Fig. 5A). In terms of Nrf2 pathway, AMPK inhibition eliminated metformin-induced Nrf2 nuclear import and binding with ARE, resulting in HO-1 protein decrease (Fig. 5B–C and sFig. 5). The previously observed mitigation in Pb-induced mitochondrial fission by metformin was not detected in lack of AMPK activation (Fig. 5D–F). Moreover, compared to treatment of metformin solely, the application of Compound C increased Drp1 phosphorylation (Fig. 5G). These results demonstrate the capacity of metformin to activate Nrf2 and mitigate Pb-induced mitochondrial fragmentation relying on AMPK/Nrf2 pathway.

### 3.6 Inhibition of mitochondrial fission rescued Pb-induced cell death and energy shortage

**Fig. 6:**
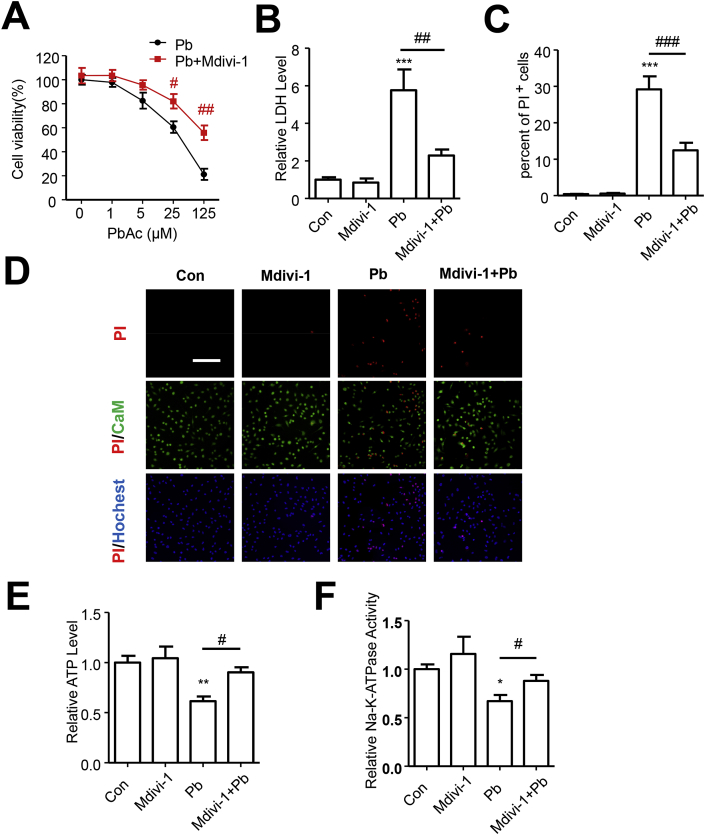
Inhibition of mitochondrial fission by Mdivi-1 rescued Pb-induced cell death and energy shortage. (A) SH-SY5Y cells pretreated with Mdivi-1 (10 μM) for 6 h were exposed to various dose of PbAc (1, 5, 25 or 125 μM) for 24 h, cell viabilities were analyzed by CCK8 assay, n = 5. (B–F) Cells were exposed to PbAc (25 μM) after treatment of Mdivi-1, LDH release in medium were detected, n = 4 (B), and CaM/PI/Hochest33342 were used to analyzed cell death (Scale bar = 500 μm, n = 6) (C–D); ATP content (E) and Na^+^-K^+^-ATPase activity (F) were detected, n = 3. *P < 0.05, **P < 0.01 and ***P < 0.001 represent significant differences compared with the untreated cells and #P < 0.05, ##P < 0.01 and ###P < 0.001 represent significant differences between groups with or without metformin pretreatment exposed to PbAc.

The defects in mitochondrial fusion and fission coordination impaired mitochondrial function, which subsequently decides cell fate [34]. To identify the contribution of boost in mitochondrial fission to Pb-induced cell death and energy shortage, Mdivi-1, a widely used inhibitor of mitochondrial fission, was introduced to investigate the role of mitochondrial fission in Pb-induced cell damage. After Mdivi-1 treatment, the decreased cell viability was elevated and the LDH leakage in medium was inhibited (Fig. 6A–B). The percent of PI^+^ cells also dramatically declined by Mdivi-1 treatment when cells were exposed to PbAc (Fig. 6C–D). Inhibition of mitochondrial fission could also suppress the PbAc-caused descent of cellular ATP level, and then elevate the Na^+^-K^+^-ATPase activity (Fig. 6E–F). Thus, Pb-induced cell death and energy shortage may partially rely on mitochondrial fragmentation.

## 4 Discussion

In the current study, we detected mitochondrial morphology and observed a vivid increase in mitochondrial fragmentation in Pb-exposed SH-SY5Y cells. The fragmentation of mitochondria is oxidative stress dependent, resulting in energy shortage and cell death. After applying metformin, Pb-induced neurotoxicity could be ameliorated by inhibiting mitochondrial fragmentation. The protective effect of metformin is mediated by activation of antioxidant Nrf2/ARE pathway in an AMPK dependent manner. Metformin is capable of abolishing Pb-induced mitochondrial fragmentation and following impairment in cell homeostasis, depending on the activation of AMPK/Nrf2 pathway.

Normal mitochondrial fission is necessary for energy production and damaged mitochondria removing. However, excessive fission, called fragmentation, would lead to oxidative phosphorylation imbalance, oxidative stress and even cell death [35,36]. Mitochondrial fragmentation caused by Pb has been reported [5]. Current results also suggested that upon Pb exposure, mitochondrial fragmentation and p-Drp1 (s616) enhancement were stimulated in a ROS dependent manner, resulting in ATP decrease and cell death. Drp1 is an important fission associated protein [7]. An investigation in Pb-triggered defects in mitochondrial dynamics revealed the regulatory effect of PGC1α on Drp1 expression [5], manifesting only the increase in level of p-Drp1 but not Drp1 or mRNA. It has been suggested that abnormal posttranslational modifications of Drp1 accelerate excessive mitochondrial fragmentation, which is associated with neuronal dysfunction and cell death [10,37]. Mdivi-1, a small molecule inhibitor to block Drp1 recruitment to mitochondria, could attenuate Pb-induced cell death and energy shortage, suggesting that Pb-triggered mitochondrial fragmentation has detrimental impact. Evidence suggested that inhibition of Drp1 activity resulted in reduced mitochondrial fission and neurotoxicity [38], along with suppressed Bax translocation to mitochondrial and cytochrome c release, which has a pivotal role in apoptosis [39]. Thus, mitochondrial fragmentation might partially mediate Pb-induced neurotoxicity.

Redox state is meticulously regulated to avoid cellular disorder, where oxidative stress is proven to disturb the homeostasis in mitochondrial dynamics and biogenesis [40,41]. Previous studies confirmed that acute exposure to H_2_O_2_ elicited oxidative stress and promoted subsequent mitochondrial fragmentation in human endothelial cells and skeletal muscle myoblasts, accompanied with enhanced activity of Drp1 [42,43]. The accumulation of ROS in cells could increase intracellular cytosolic Ca^2+^ concentrations required for mitochondrial fission by triggering the ROS-sensitive transient receptor potential channel, subtype melastatin 2 (TRPM2) ion channels open during the high glucose treatment in human umbilical vein endothelial cells (HUVECs) [44]. In consistent with these results above, current results suggested that oxidative stress played a role in Pb-triggered mitochondrial fragmentation, which could be attenuated by NAC. Pb-induced ROS production originated mainly from mitochondria in SH-SY5Y cells by inducing mitochondrial permeability transition and disturbing oxidative phosphorylation [26]. ROS accumulation and mitochondrial fragmentation could promote each other mutually, finally resulting in cell death, while antioxidants or mitochondrial fission inhibitors, like Mdivi-1, cut off this cycle to facilitate cell return to normal. Thus, oxidative stress and mitochondrial fragmentation are both promising targets to prevent Pb neurotoxicity.

Metformin, a universally acknowledged anti-diabetes drug and AMPK activator, has been found to be protective against Pb-induced nephrotoxicity in rat [27]. Consistently, we also detected a blunted induction in Pb-triggered mitochondrial fragmentation phosphorylation on Drp1 serine 616 in neurons upon metformin treatment. It is suggested that in the maintenance of mitochondrial dynamics, AMPK pathway plays a crucially regulatory role. Several studies found that ROS-related mitochondrial fragmentation was attenuated by pharmacological activation of AMPK in endothelial cells [45]. Researchers further elucidate the linkage by detecting the increased level of phosphorylated Drp1 at serine 637, manifesting suppressed Drp1 activity [45,46]. Another tantalizing link between metformin and inhibited mitochondrial fragmentation was proposed that metformin facilitated Drp1 degradation through autophagy-lysosome pathway [15]. However, several studies proposed the favorable effect of AMPK activation on the execution of mitochondrial fission, which was dependent on mitochondrial fission factor activation, a receptor for Drp1 [13]. In the present study, metformin inhibited Pb-induced mitochondrial fragmentation via Drp1 suppression. Such inconsistency may be partially explained by the different dose of metformin applied and different research objects. Here, we detected a marked protective role of metformin at 2 mM against Pb-induced cell damage, which may be mediated by the antioxidative effect of metformin.

Administration of metformin ameliorated ROS accumulation and GSH decline, which resulted in suppressed mitochondrial fragmentation, implicating the potential involvement of antioxidant response. This antioxidative effect of metformin originated from the activation of Nrf2-ARE system, an extremely important antioxidant detoxification system in organism, as knockdown of Nrf2 resulted in crippling the protection against Pb-induced mitochondrial fragmentation. These results are consistent with others. Zimmermann et al. observed a dampened XN-triggered Nrf2/HO-1 signaling axis in AMPK knockout cell [32]. Another study indicated that Rifampicin-induced Nrf2 translocation to nucleus was mediated by AMPKα [47]. The molecular explanation for this response has been proposed to involve glycogen synthase kinase 3β and P62 [48,49]. Recently, it has been revealed that activated AMPK directly favored phosphorylation of Nrf2 at serine 374, 408 or 433 [24]. As a redox sensor, Nrf2 is activated to upregulate its downstream antioxidant protean, for instance, HO-1 in defense of oxidative stress. Our previous work has confirmed that Pb-induced elevated ROS production and cell death could be alleviated by Nrf2/HO-1 activation [25]. Here, by knocking down Nrf2 expression, we demonstrated that AMPK/Nrf2 activation mediated the protection of metformin against Pb-induced mitochondrial fragmentation and the following impairment. Besides AMPK-dependent pathway, metformin could alleviate oxidative stress via AKT, IDH1 pathway, or block ROS generation by targeting mitochondrial respiratory-chain complex 1 [50,51]. Both AMPK dependent and independent effects may co-exist in cells. However, investigation of AMPK–independent pathways was not included in the present study, and should be implemented by future studies. Though many studies have confirmed the antioxidative effect of metformin, there are still contradictory conclusions that metformin induces ROS production to confer antiproliferative effect, especially in cancer cells [52]. The administration of metformin is proposed to stimulate metabolic stress, possibly through indirect mechanisms, for example, by inhibiting oxidative phosphorylation enzymes to disturb energy supply [52]. The inconsistency may due to various degree of tolerance for energy status from different cell types [53], and the conflicting results still warrant evidence to illustrate. Moreover, current results were acquired only in one cell line. Further investigation in vivo should be performed to examine the protective effect of metformin against Pb.

In conclusion, Pb exposure induced ROS accumulation, resulting in mitochondrial fragmentation, energy shortage and cell death. Metformin exerts a protective effect against lead toxicity, dependent on the activation of AMPK/Nrf2 signaling pathway. On Pb exposure, metformin acts as a mitochondrial fragmentation inhibitor and attenuates Pb-induced cell damage, where the suppression of Drp1 activity is involved. These findings support that metformin, serving as a mitochondrial fragmentation inhibitor, exerts protective effect against Pb toxicity. To maintain the balance in mitochondrial dynamics might be a potential target to facilitate the elimination of Pb-induced neuron injury.

## Author contributions

Jun Chen, Wei Chang and Fang Ye conceived the project and designed experiments; Luoyao Yang, Xiaoyi Li, Anli Jiang and Xintong Li performed the experiments; Fang Ye analyzed the data; Luoyao Yang wrote the manuscript; Jun Chen assisted in data interpretation and edited the manuscript.

## Declaration of competing interest

The authors declare that they have no known competing financial interests or personal relationships that could have appeared to influence the work reported in this paper.
