# Supplementary material for: Metformin alleviates lead-induced mitochondrial fragmentation via AMPK/Nrf2 activation in SH-SY5Y cells
Source: Redox Biol. 2020 Jun 30;36:101626. doi: 10.1016/j.redox.2020.101626 (PMC7334619; doi:10.1016/j.redox.2020.101626)
Supplement: Multimedia component 1 [file mmc1.docx]

**S table 1**. Antibody used in current study

| Antibody | Source | Identifier |
| --- | --- | --- |
| AMPK | Cell Signaling Technology | 5831 |
| Drp1 | Cell Signaling Technology | 8570 |
| HO-1 | Abcam | ab13248 |
| Mfn1 | Proteintech | 13798-1-AP |
| Nrf2 | Santa Cruze | sc-365949 |
| p-AMPK | Cell Signaling Technology | 50081 |
| p-Drp1 | Cell Signaling Technology | 4494 |
| P-Raptor | Cell Signaling Technology | 2083 |
| Raptor | Cell Signaling Technology | 2280 |
| Tom20 | Santa Cruze | sc17764 |
| β-actin | Abbkine | A01010 |
| Alexa Fluor 488 | Invitrogen | A-11029 |

**S Figure 1**

**
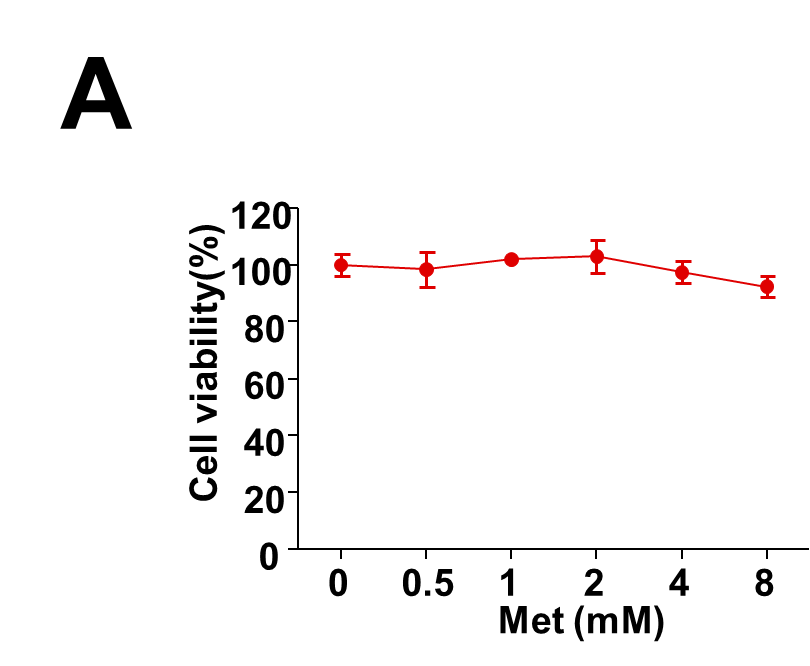
**

**S Figure 1.** Metformin did not induce reduction of cell viability. SH-SY5Y cells were treated with various dose of metformin (0.5, 1, 2, 4 or 8mM), cell viability was analyzed by CCK8 assay, n=5.

**S Figure 2**

**
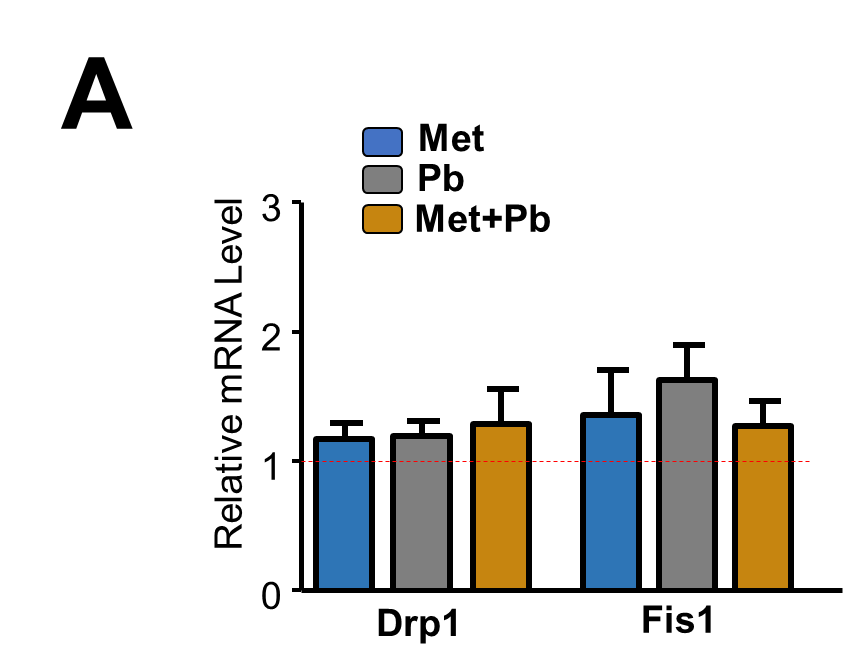
**

**S Figure 2.** Metformin did not induce activation of Drp1 and Fis1. After treatment, the mRNA level of the fission associated proteins (Drp1 and Fis1) were analyzed by RT-PCR, n=3.

**S Figure 3**

**
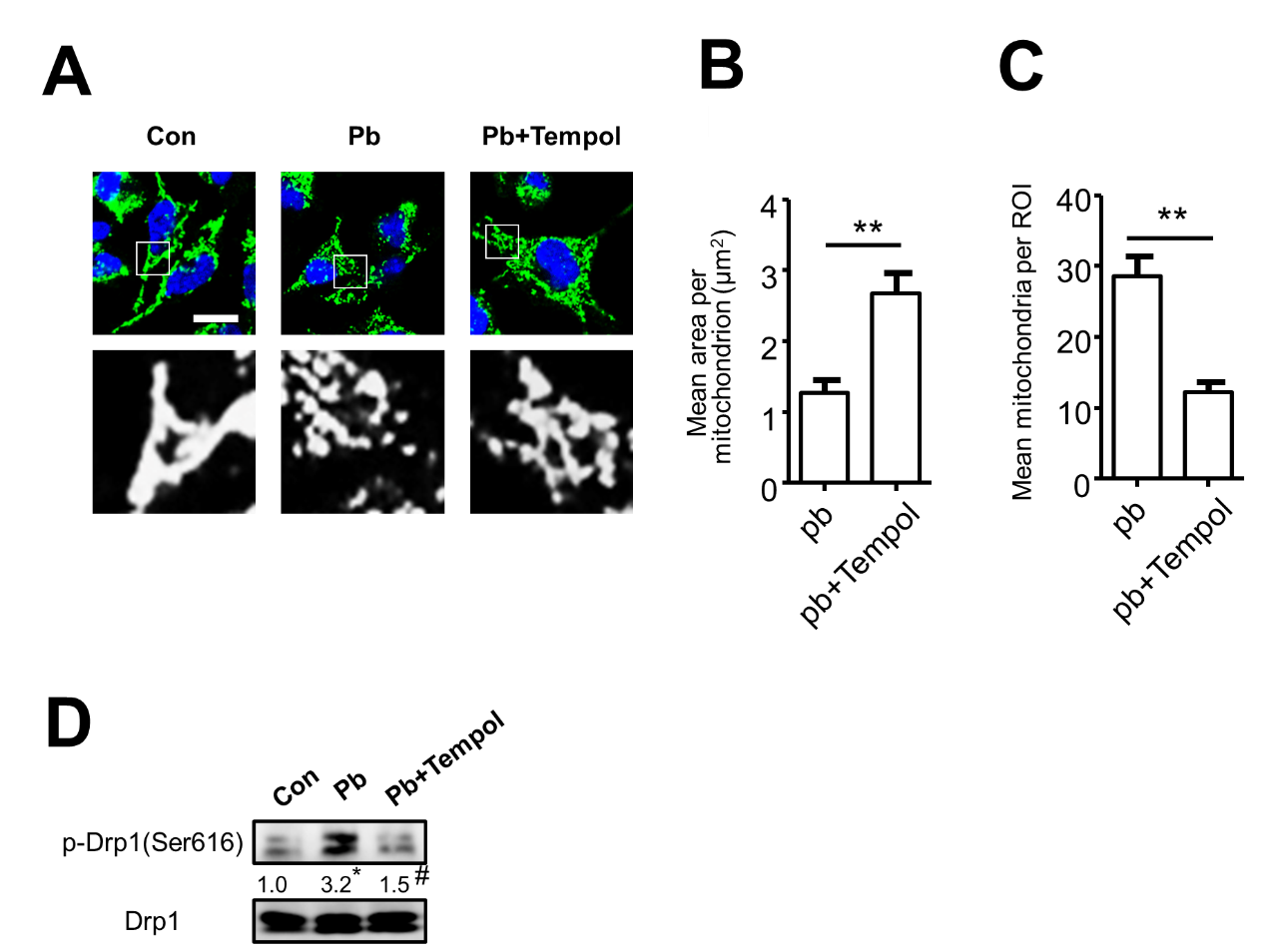
**

**S Figure 3.** Tempol inhibited PbAc-induced mitochondrial fragmentation. (A-D) SH-SY5Y cells pretreated with Tempol (0.5mM) were exposed to PbAc for 24h, the immunofluorescence of TOM20 were performed and the mitochondrial morphology was analyzed, scale bar=10μm, n=25 (A-C); the levels of p-Drp1 (s616) and Drp1 were detected, n=3 (D). * P<0.05 and ** P<0.01 represent significant differences compared with the untreated cells and # P<0.05 represents significant differences between groups with or without Tempol pretreatment exposed to PbAc.

**S Figure 4**

**
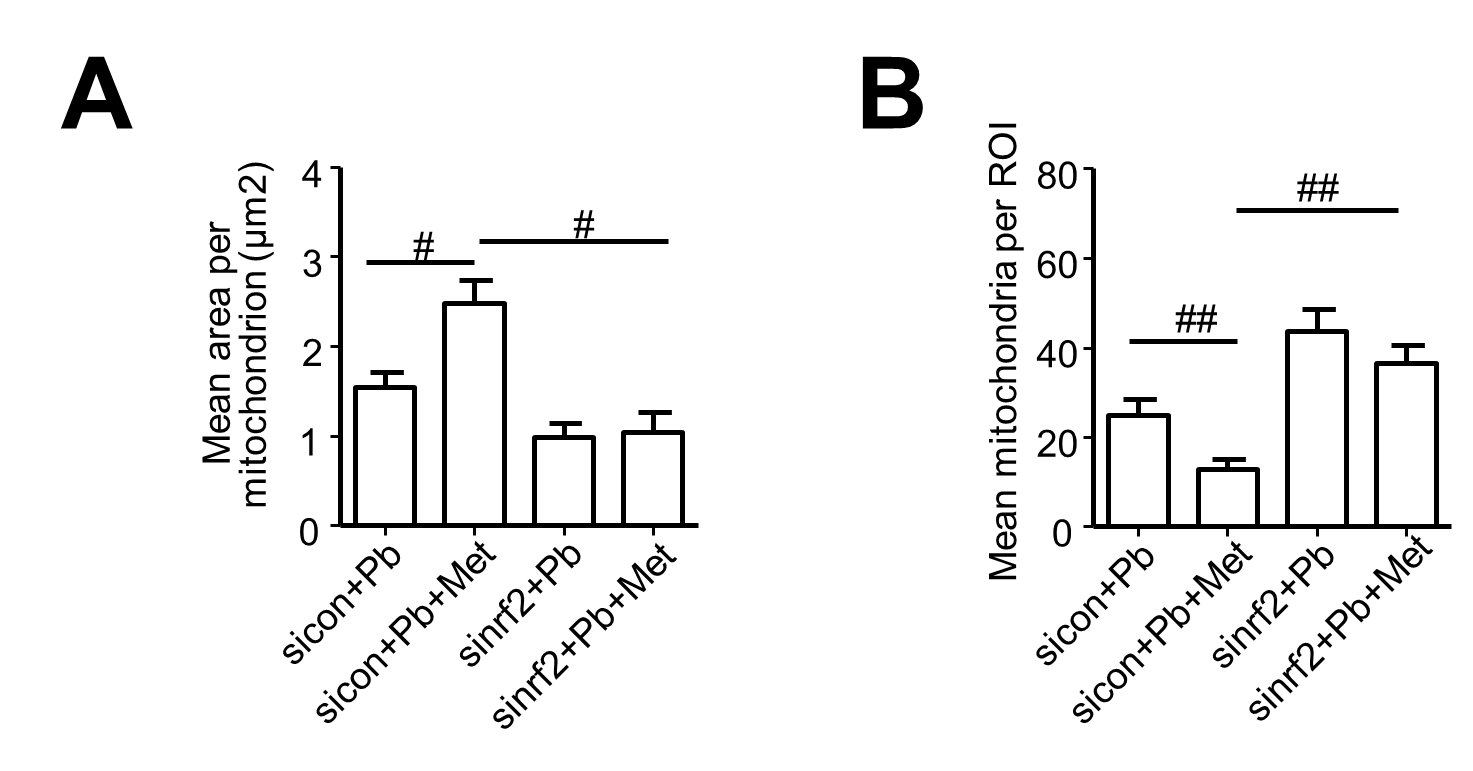
**

**S Figure 4.** Supplement to Figure 4G.

**S Figure 5.**

**
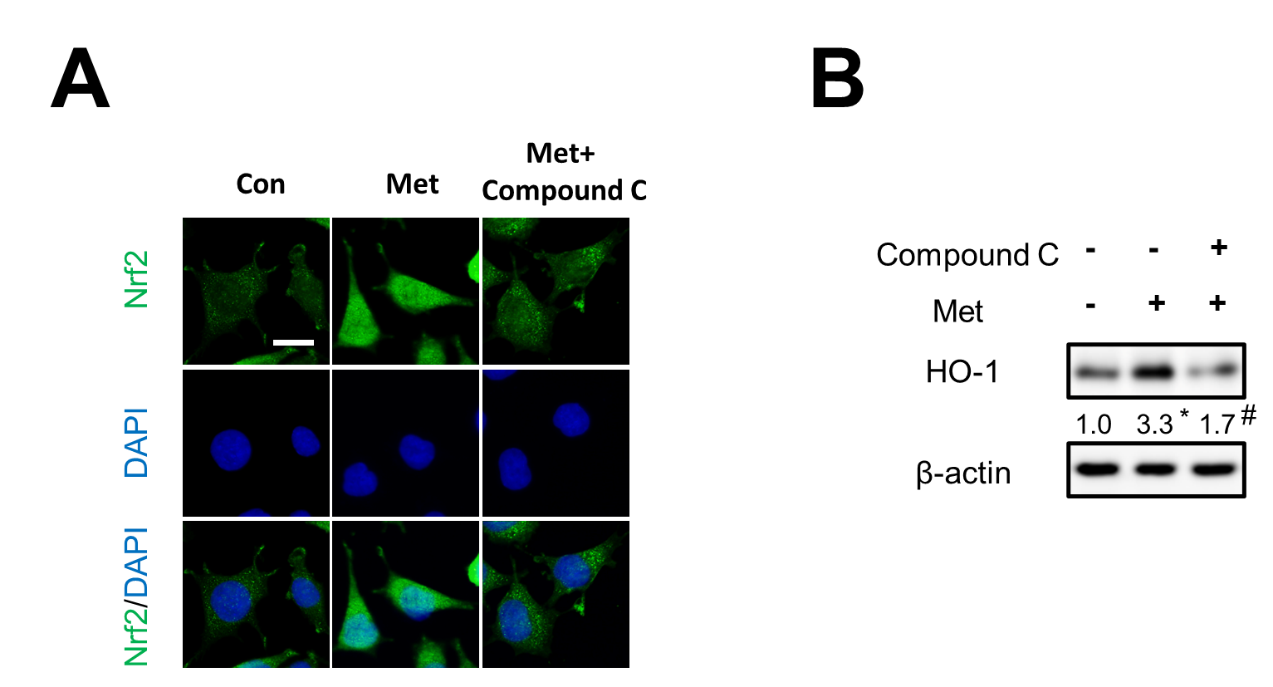
**

**S Figure 5.** compound C inhibited Nrf2/HO-1 activation caused by metformin treatment.SH-SY5Y cells were treated with compound C (10μM) and metformin for 24h, immunofluorescence of Nrf2 was performed, scalebar= 10μm (A); western blot detected the protein of HO-1, n=3 (B).
